# 

**Published:** 1893-10-21

**Authors:** 


**OUR NEW OFFICES,**
428, Strand, W.C., will henceforth be the Office of this Journal.

**Notices of Births, Deaths, and Marriages** are inserted in this column at a charge of 2s. 6d. for an announcement not exceeding four lines.

### NOTICE TO CORRESPONDENTS.

All MS., Letters, Books for Review, and other matters intended for the Editor should be addressed The Editor, The Lodge, Porchester Square, London, W.

The Editor cannot undertake to return rejected MS., even when accompanied by stamped directed envelope.

**Notice to Advertisers and Subscribers.**

All Advertisements, Orders for Copies of The Hospital, and Business Communications should be addressed to The Publisher, *not to the Editor*, The Hospital, 428, Strand, W.C.

The Cost of Subscription, post free, is as follows:—Per week, 2½d.; per quarter, 2s. 6d.; per half-year, 5s.; per annum, 8s. 6d Foreign:—Per Annum, 10s. 8d.; payable, in all cases, in advance.

**"Burdett's Hospital Annual," 1893.**

Fourth year of publication. 700 pp. Boards, gilt lettering. A most complete guide to British, Colonial, Indian, and American Hospitals, Dispensaries, Nursing and Convalescent Institutions, and Asylums. Price 5s. Published by The Scientific Press (Limited), 428, Strand, W.C.

**NOTICE.—The Index for Vol. XIII. (ending March, 1893) is on sale at the Office of this Journal, price 2½d., post free.**

### Scraps and Gleanings.

The sum of £40 has recently been received by the Worcester Infirmary in new and additional subscriptions.

An anonymous donation of £500 has been sent to the treasurer of Guy's Hospital for the benefit of the institution.

The number of in-patients treated at the Stockton Hospital during the past year was 83, and there were 243 out-patients.

The people of Tunbridge Wells were very energetic in their demands on Hospital Saturday, and collected the goodly sum of £65.

A French hospital will shortly be erected at Brussels, the site selected for the new building being close to the Royal Park.

The International Association of Women Pharmacists in America has now 709 members; their first congress was lately held at Chicago.

It has been proposed that the commercial travellers of Glasgow should organise a demonstration in aid of the local hospitals and life-boats.

A new eye infirmary, which has been erected at a cost of £7,000, was opened at Sunderland on October 16th by the Marchioness of Londonderry.

The first Hospital Tuesday held in Ipswich took place recently with most encouraging results, £180 being collected for the East Suffolk Hospital.

For the benefit of the Prince Alfred Hospital, at Sydney (N.S.W.), a "self-denial" fund was started in the town, which realised £3,000 towards the institution.

Some indignation has been caused by the action of the Board of the Belvidere Fever Hospital, Glasgow, in refusing to take patients from ratepayers staying in the country.

A meeting of the ratepayers of Forfar was recently held to protest against the addition of infectious diseases wards to the Forfar Infirmary as dangerous to the inhabitants of the town.

Watford distinguished itself on Hospital Saturday by energy, resource, and success. Stalls were erected throughout the town, and the labours of the day resulted in the large sum of £130 in collections.

It has been decided to again hold two Cinderella dances during the coming season owing to the unprecedented success of the couple held last winter. The profits go to the Chelsea Hospital for Women.

An Infectious Diseases Hospital is urgently needed for Houghton District, Northumberland. Cases both of scarlet fever and typhoid have broken out, and at present no efficient means of isolation exist.

The Local Boards of Marsden, Staithwaite, Golcar, and Linthwaite, have agreed to amalgamate for the purpose of carrying on the infectious diseases hospital at Moor Top, which is now in full working order

The annual report of the governors of the Lowestoft Hospital shows an expenditure £40 in excess of last year, caused by the large increase of out-patients, while in-patients and working expenses have been reduced. The hospital has been recently enlarged.

The Committee of the Adelaide Hospital, Dublin, are to be congratulated on the satisfactory report just issued. The hospital now contains 135 beds, and 1,248 cases were treated during the past year, the patients remaining about a month in the institution.

An unusual amount of opposition has been aroused by the proposal to erect an infectious diseases Hospital at Shooters Hill. The supporters of the scheme contend that the proposed site is an eminently suitable one, and presents no danger to the neighbourhood.

It is much to be regretted that no site has yet been decided upon for the Leicester Infectious Diseases Hospital. During the last two months 133 cases of diphtheria, and over 1,000 of scarlet fever have occured, while nearly all the available accommodation has been used for small-pox cases.

The Lord Mayor has distributed the fruit recently presented to him by the Fruiterers' Company among the patients of the North-Eastern Hospital for Children, the Royal Hospital for Children and Women, Waterloo Bridge Road, and the Royal Hospital for Consumption, City Road.

At a recent meeting of the Municipal Council of the City of Dublin considerable excitement was caused by the proposal of the Hospitals Committee to withdraw the grants from the Combe and Rotunda Hospitals on the ground of their alleged inefficiency. No decision was arrived at.

An appeal has been issued by the Committee of the Swansea Provident Dispensary for donations to wipe out the debt of £80, and for increased annual subscriptions. Members' payments amount to £650 per annum, and an additional £80 in subscriptions would be sufficient to support the institution.

A proposal has been laid before the Metropolitan Asylums Board to purchase an additional acre of land for the site already procured for the Fountain (temporary) Infectious Diseases Hospital, as the present acreage only provides sufficient room for a hospital with 350 beds, instead of 400 as intended.

### Books Received.

Macmillan and Co.

"A Text Book of General Therapeutics." By W. Hale White, M.D., F.R.C.P.

"Handbook of Public Health and Demography." By Edward F. Willoughby, M.D.

Edward Stanford.

"Epidemics, Plagues, and Fevers; their Causes and Prevention." By Hon. Rollo Russell.

E. Livingstone, Edinburgh.

"The Student's Handbook of Gynecology."

H. K. Lewis.

"A Guide to the Examination of Urine." By J. Wickham Legg, F.R.C.P.

The Clarendon Press.

"Hospital Construction." By Sir Douglas Galton.

A. D. Innes and Co.

"Ladies at Work." By Experts in several branches.

**Periodicals and Pamphlets.**—The Young Gentlewoman, The Leisure Hour, The Sunday at Home, The Girl's Own Paper, The Boy's Own Paper, The Therapeutic Gazette, "The Stæchiological Cure of Consumption," by J. F. Churchhill, M.D. Birmingham Medical Review, The Charity Organisation Review, The Religious Review of Reviews, The Review of Reviews, The Athenæum, The Academy, The Westminster Review, London, The Homœopathic Review, The Homœopathic World, Tit Bits, The Strand Magazine, The Picture Magazine, The Clinical Journal, Reich's Medicinal, Anzeiger, Guy's Hospital Gazette, Toilers of the Deep, Report of Bath and Somerset Pauper Lunatic Asylum, The Medical Chronicle, Zenana Mission Paper, The English Illustrated Magazine.

### MEDICAL VACANCIES AND APPOINTMENTS.

### APPOINTMENTS.

F. J. Allan, M.D.Edin., appointed Extra-professional Examiner in Medical Jurisprudence and Public Health for Degrees in Medicine at the University of Aberdeen. W. B. Betenson, L.R.C.P. Lond., M.R.C.S.Eng., appointed Medical Officer for the Fourth District of the London and Clavering Union. J. Bostock, M.R.C.S.Eng., L.S.A., reappointed Medical Officer for the Wymeswold District of the Loughborough Union. R. E. Brown, L.R.C.P., L.R.C.S.Edin., appointed Medical Officer at the Willington Station of the North-Eastern Railway Company. J. W. Byers, M.A., M.D., appointed Professor of Midwifery and Diseases of Women and Children in Queen's College, Belfast. S. Coupland, M.D.Lond., appointed Extra-professorial Examiner in Medicine for Degrees in Medicine at the University of Aberdeen. Andrew Croll, M.B., C.M., Resident Clinical Assistant, Dundee Royal Lunatic Asylum, appointed Medical Officer Dundee Poorhouses. J. S. Ferris, M.B., L.R.C.P.Lond., M.R.C.S.Eng., reappointed Medical Officer and Public Vaccinator for the Denham Sanitary District of the Eton Union. R. J. H. Gibson, M.A. Aberd., appointed Extra-professorial Examiner in Botany for Degrees in Medicine at the University of Aberdeen. A. S. Gubb, M.D., appointed Medical Officer to the National Telephone Company (Metropolitan Division). J. Hunter, F.I.C., F.C.S.Edin., appointed Extra-professorial Examiner in Chemistry for Degrees in Medicine at the University of Aberdeen. W. R. Y. Ives, L.R.C.P.Lond., L.S.A., appointed Surgeon to the Police Force of the Portswood District. J. Johnston, M.D., C.M.Edin., L.S.A. Lond., appointed Honorary Surgeon to the Bolton Infirmary and Dispensary. W. C. M'Donnell, L.R.C.P.Lond., M.R.C.S.Eng., appointed Medical Officer for the Sixth District of the Hackney Union. F. R. Mallett, M.B., C.M.Edin., L.R.C.P., L.R.C.S. Edin., appointed Honorary Surgeon to the Bolton Infirmary and Dispensary. R. D. Mothersole, M.D., M.S.Lond., F.R.C.S.Eng., L.R.C.P.Lond., appointed Honorary Surgeon to the Bolton Infirmary and Dispensary. W. P. O'Meara, L.R.C.P., L.R.C.S.Edin., appointed Surgeon to the Police Force of Southampton. H. O. Pilkington, M.R.C.S.Eng., reappointed Medical Officer of Health for the Preston Urban Sanitary District and for the Port Sanitary Authority. J. L. Smith, M.A., M.D.Edin., appointed Extra-professorial Examiner in Pathology for Degrees in Medicine at the University of Aberdeen. G. N. Stewart, M.D.Edin., appointed Extra-professorial Examiner in Physiology for Degrees in Medicine at the University of Aberdeen. J. A. Thomson, M.A.Edin., appointed Extra-professorial Examiner in Zoology for Degrees in Medicine at the University of Aberdeen. E. T. Thompson, L.R.C.P., L.R.C.S.I., reappointed Medical Officer and Public Vaccinator for the Dunstable District of the Luton Union. M. A. Ward, M.D.Dub., F.R.C.S.I., appointed Medical Officer of Health for Rathmines, co. Dublin. F. Warner, M.D.Lond., appointed Extra-professorial Examiner in Materia Medica for Degrees in Medicine at the University of Aberdeen. R. H. Wellington, M.R.C.S., L.R.C.P.Lond., reappointed Medical Officer of Health for the Sutton Urban Sanitary District, and to the Port of Wisbech, and Medical Officer to the Port Hospital.

### VACANCIES.

The following vacancies are announced this week, the amount of salary in each case being given after the name of the institution:

**Resident Medical Officers.**

Brixton, Streatham, and Herne Hill Dispensary, Water Lane, Brixton. Resident House Surgeon; unmarried; doubly qualified. Salary £150 per annum, with furnished apartments, attendance, coal, and gas. Applications to the Secretary by October 27th.

Dundee Royal Lunatic Asylum. Resident Clinical Assistant. Application to Dr. Rorie, Westgreen House, Dundee.

General Hospital, Birmingham. Two Assistant House Surgeons. Residence, board, and washing provided. Applications to the House Governor by October 28th.

Huddersfield Infirmary. Senior House Surgeon. Salary £80 per annum, with board, lodging, and washing. Applications to Mr. Joseph Bate, Secretary, by October 23rd.

**Non-Resident Medical Officers.**

Great Northern Central Hospital, Holloway Road, N. Surgeon to the out-patients; must be F.R.C.S.Eng. Applications to William T. Grant, Secretary, by October 23rd.

Hospital for Sick Children, Great Ormond Street, W.C. Surgical Registrar and Anæsthetist. Appointment for one year, an honorarium of £40 being voted at the expiration of that term. Applications to the Secretary by October 24th.

Parish of St. Mary, Islington. Medical Officer for the Third District. Salary £100 per annum, with extra fees. Applications to Edwin Davey, Clerk, Guardians' Office, St. John's Road, Upper Holloway, N., by October 24th.

West London Hospital, Hammersmith Road, W. Physician. Must be Fellow or Member of the Royal College of Physicians of London. Applications to R. J. Gilbert, Secretary-Superintendent, by October 25th.

### UNIVERSITY COLLEGE, LONDON.

### FACULTY OF MEDICINE.

Report of the Dean for the Session 1892-93.

The number of students who have attended the classes of the Faculty during the past session is 340, 113 of whom were new students. Not only do these numbers compare favourably with those of some former years, but the standard of excellence which has always distinguished the work of this medical school has been more than maintained, as the following results of the University of London examinations conclusively show: Of 64 who took the degree of M.D., 18 were from University College; of 87 who took the degree of M.B., 22 were from University College; of 33 who took the degree of B.S., 7 were from University College; and of 156 who passed the Int.M.B., 39 were from University College. Thus, out of a total of 340 who passed the various medical examinations of the University, 86 were from University College.

The results of the Honours examinations point still more conclusively to the same fact.

At the M.D., Messrs. P. R. Dodwell, H. M. Richards, and W. M. Stevens obtained the number of marks qualifying for the Gold Medal.

At the M.B. Examination last November, Mr. H. A. Ballance obtained First-Class Honours with marks qualifying for the Gold Medal in Medicine, and First-Class Honours in Midwifery. Mr. J. Evans obtained Honours in Medicine. Mr. A. D. Heath obtained Honours in Medicine. Mr. J. Jones obtained First-Class Honours in Forensic Medicine. Mr. C. B. T. Musgrave obtained Honours in Medicine. Mr. M. Randall, B.A., obtained First-Class Honours in Medicine. Mr. H. R. Smith obtained First-Class Honours in Medicine. Mr. C. G. Spencer obtained Honours in Medicine, Obstetric Medicine and Forensic Medicine. Mr. G. A. Stephens, B.Sc., obtained Honours in Medicine.

At the B.S. Examination, Mr. H. A. Balance obtained the Gold Medal. Mr. J. L. Firth, M.D., obtained Honours.

At the Intermediate M.B. Examination in July last, Mr. J. H. Cook obtained First-Class Honours in Physiology, and Honours in Materia Medica. Mr. A. Dimsey obtained the Gold Medal in Physiology, First-class Honours in Materia Medica, and Hounours in Anatomy. Mr. A. J. Rodocanachi obtained the Exhibition and Gold Medal in Organic Chemistry. Mr. J. D. Russell obtained Honours in Anatomy and Physiology. Mr. H. B. Shaw obtained the Exhibition and Gold Medal in Anatomy and Honours in Physiology. Mr. T. H. C. Stevenson obtained Honours in Anatomy.

At other Universities the following degrees have been obtained by our Students:—

University of Cambridge—Diploma in Public Health, Messrs. E. M. Wilson and M. G. Yunge-Bateman.

At the University of Durham—M.D., Dr. E. B. Hulbert; and M.B., Mr. S. A. Leigh-Sodipo.

The results of Examinations for Diplomas are as follows: Three gentlemen obtained the Diploma of F.R.C.S., 36 gentlemen obtained the Diploma of M.R.C.S., 36 gentlemen obtained the Diploma of L.R.C.P., 5 gentlemen obtained the Diploma of M.R.C.P., 5 gentlemen obtained the Diploma of Public Health.

It is with deep regret that I record the loss which our school has sustained in the untimely death of Mr. Marcus Beck, Professor of Surgery and of Clinical Surgery, who, as a student and teacher, was connected with the college for nearly thirty years. The vacancies caused by Mr. Beck's death have been filled by the appointment of Mr. Barker to the Professorship of Surgery and Mr. Godlee to the Professorship of Clinical Surgery, while Mr. Raymond Johnson has been appointed Assistant Surgeon to the Hospital.

Dr. John Williams, Professor of Midwifery since 1887 and Obstetric Physician to the Hospital, has, to the regret of his colleagues, found it necessary to resign those appointments, and has been succeeded by Dr. H. Spencer; and Dr. G. F. Blacker has been appointed Assistant Obstetric Physician. In connection with these changes I may record with an expression of sincere regret the death of Dr. Graily Hewitt, which has occurred since the close of the Session in July. Dr. Hewitt formerly occupied the posts which have just been vacated by Dr. John Williams, and his teaching has influenced many generations of our students.

This report would be incomplete without some notice of the large amount of original research which has been carried on for many years in the Laboratories of this Faculty, and which has greatly increased of late in consequence of the establishment of the new Laboratory of Pathology.

E. A. SCHAFER, *Dean.*

### The Hospital Advertiser.

### MEDICAL APPOINTMENTS.

THE HOSPITAL FOR SICK CHILDREN, GREAT ORMOND STREET, W.C.

